# Central Serous Chorioretinopathy Risk Factors in An Iranian Cohort: A Case-control Study

**DOI:** 10.18502/jovr.v18i4.14553

**Published:** 2023-11-30

**Authors:** Saeed Karimi, Nastaran Payandeh, Sahar Mahmoudi Nejad Azar, Hosein Nouri, Seyed-Hossein Abtahi

**Affiliations:** ^1^Ophthalmic Research Center, Research Institute for Ophthalmology and Vision Science, Shahid Beheshti University of Medical Sciences, Tehran, Iran; ^2^Department of Ophthalmology, Torfeh Medical Center, Shahid Beheshti University of Medical Sciences, Tehran, Iran; ^3^Clinical Research Development Unit of Torfeh Medical Center, Shahid Beheshti University of Medical Sciences, Tehran, Iran; ^4^School of Medicine, Shahid Beheshti University of Medical Sciences, Tehran, Iran; ^5^School of Medicine, Isfahan University of Medical Sciences, Isfahan, Iran; ^7^Saeed Karimi: https://orcid.org/0000-0002-3231-8414; ^8^Sahar Mahmoudinejad Azar: https://orcid.org/0000-0002-5124-3454

**Keywords:** Central Serous Chorioretinopathy, Risk Factors, Smoking; Alcohol Drinking, Psychological Stress

## Abstract

**Purpose:**

This study aimed to investigate the possible risk factors of central serous chorioretinopathy (CSCR) in an Iranian cohort.

**Methods:**

We recruited 39 consecutive CSCR cases and 80 age-matched controls with no ocular pathology from the same medical center from March 2019 to March 2021. Enrolled patients underwent complete ophthalmological examination and extensive history taking in a referral setting. Logistic regression analysis was performed to detect any possible association of potential risk factors with CSCR.

**Results:**

The mean age of CSCR patients was 40.69 ± 7.71 years. The male-to-female ratio in cases and controls was 1:1.79 and 1.22:1, respectively. Logistic regression analysis revealed that alcohol consumption (odds ratio, OR: 62.0, P< 0.001), smoking (OR: 4.0, 95% CI: 1.47-10.85, P< 0.006), corticosteroid use (OR: 6.95, 95% CI: 2.64-18.28, P< 0.001), and high psychological stress (OR: 13.34, 95% CI: 4.68-38.02, P< 0.01) were significant risk factors for developing CSCR. Ischemic heart disease ( P =0.550), hypertension ( P =0.750), and hyperopia ( P =0.467) were not associated with the risk of CSCR. The most common form of steroid use was oral in both groups. No pregnant female was present in the study sample, precluding the assessment of its potential association with CSCR.

**Conclusion:**

CSCR often affects individuals of productive working ages; thus, identifying its preventable risk factors is highly encouraged. Our results suggested that alcohol consumption, smoking, and high levels of psychological stress are independent, preventable risk factors for CSCR.

##  Introduction

The fourth most common non-surgical retinal disease, central serous chorioretinopathy (CSCR), is characterized by altered choroidal permeability, choroidal thickening, and retinal pigment epithelium (RPE) dysfunction resulting in subretinal fluid accumulation, neurosensory retinal detachment, and visual disturbance^[1,2]^. Visual complaints include central vision loss, scotoma, micropsia, or metamorphopsia^[3]^. CSCR is usually idiopathic and resolves spontaneously within four months, but it can have severe visual impacts, such as retinal dysfunction and permanent visual loss^[2,4]^. CSCR most commonly affects young to middle-aged men between the ages of 30 to 50. As the disease affects patients in their productive working years, more data on preventable predisposing factors are required to help plan efficient preventive strategies^[2]^.

Despite extensive research, the exact pathophysiology of CSCR remains hypothetical^[1]^. However, increased hydrostatic pressure in the thickened choroid and increased permeability of the choroidal vessels may contribute to RPE barrier dysfunction. Such dysfunction may allow fluid leakage into the subretinal space and, consequently, neurosensory retinal detachment^[5,6]^. The most commonly reported possible risk factors for CSCR include age, male gender, high levels of corticosteroids, psychological stress, H. pylori infection, systemic hypertension, history of cardiovascular diseases, obstructive sleep apnea, abnormal coagulation or platelet aggregation, smoking, alcohol consumption, hyperopia, and pregnancy^[4,7,8,9,10]^. The association of some of these factors (e.g., smoking) with the disease have been inconsistent^[1,4,11,12]^. This study aimed to investigate the association of possible risk factors with CSCR among the Iranian population.

##  Methods

This case-control study was conducted at a tertiary eye care center in Tehran, Iran. The protocol of the study was approved by the Ethics Committee of Shahid Beheshti University of Medical Sciences, Tehran, Iran (IR.SBMU.MSP.REC.1399.674) and followed the tenets of the Declaration of Helsinki. Consecutive patients older than 18 diagnosed with acute or chronic CSCR in the study center from March 2019 to March 2021 were enrolled. CSCR was defined as a localized neurosensory retinal detachment associated with a focal leak at the RPE level. Symptomatic cases with subretinal fluid for more than six months were considered “chronic” CSCR. The diagnosis was based on clinical fundus examination, optical coherence tomography (OCT), and fluorescein angiography (FA) findings.

Patients with other related ocular conditions such as uveitis, optic disc edema, choroidal infiltration, cotton wool spots, retinal hemorrhage, myopic macular pathologies, diabetic retinopathy, polypoidal choroidal vasculopathy, glaucoma, and Harada disease were excluded; so were those with history of any recent surgery or trauma within a month of presentation.

Apart from the CSCR group, a control group of patients, matched for age, who had visited our department within the same timeframe for routine examination without having any major ocular disorder was assembled. Patients with incomplete medical records (including ophthalmic history and examination, medical history, drug history, and social history) were excluded. Eligible participants were contacted, informed about the study, and asked to fill out a pre-designed questionnaire. They were also asked to consent to the use of their medical records, subject to anonymization. Additional data was collected by retrospectively reviewing patients' medical records and paraclinical documents. To confirm or rule out the diagnosis of CSCR, all patients had undergone a thorough ophthalmic examination, including best-corrected visual acuity (BCVA) measurement using the Snellen chart, slit lamp biomicroscopy, fundus examination, OCT and FA.

The questionnaire was composed of the following variables: age, gender, alcohol consumption, smoking, coronary heart disease (CHD), systemic hypertension, hyperopia, and any forms of steroid use (oral, nasal, and injection – including intravenous, intramuscular, periocular, or intravitreal routes), psychological stress, and pregnancy. All intended variables (except age and gender) were regarded in a binary format as “present” or “absent.” Concerning psychological stress, participants responded to 10 questions (within the questionnaire) according to the Perceived Stress Scale, designed by Cohen et al.^[13]^ The variable “stress” was dichotomized to simplify the statistical modeling and was considered to be “present” in patients with a score of 27/40 or more (classified as high perceived stress) and “absent” in those with a score of 0-26 (low-to-moderate perceived stress). The presence of antihypertensive drugs alone in the medication history was considered “present” hypertension. Smoking was considered “present” only if it was one pack-year or more. Steroid use – oral or nasal daily regimens or a single injection – must have been within two months before CSCR onset to be considered “present.”

The data were analyzed using IBM SPSS 25.0 software. Results were expressed as mean ± standard deviation (SD) for quantitative variables and percentages for categorical variables. The Chi-square test was used to detect inter-variable relationships and regression analysis to investigate the association of continuous variables with the presence of the disease. For all tests, a P -value of < 0.05 was considered statistically significant.

##  Results

We included 39 patients with acute ( n =15, 38.5%) and chronic ( n =24, 61.5%) CSCR in the case group and 80 age-matched subjects without any significant ophthalmic pathology in the control group. The mean age was 40.69 ± 7.71 years in the case group and 40.58 ± 9.93 in the control group ( P =0.944). Male to female ratio in cases and controls were 1:1.79 and 1.22:1, respectively ( P =0.052). The demographic and clinical data of all patients are shown in Table 1.

Logistic regression analysis showed alcohol consumption, smoking, corticosteroid use, and psychological stress were associated with CSCR. Eleven patients (28.2%) among cases and none among controls had alcohol consumption (odds ratio, OR: 62.0, P< 0.001). A history of smoking at least one pack-year was present in 12 patients (30.8%) in the case group and eight patients (10.0%) in the control group (OR: 4.0, 95 % CI: 1.47-10.85, P =0.006). Seventeen patients (43.6%; 11 oral, 4 oral + injection, and 2 oral + nasal) in the case group and eight in the control group (10.0%; 6 oral, 1 eye-drop, and 1 oral + injection) had at least one corticosteroid administration in their medication history – within two months before CSCR onset (OR: 6.95, 95% CI: 2.64-18.28, P< 0.01). Moreover, high psychological stress was similarly associated with CSCR (OR: 13.34, 95% CI: 4.68-38.02, P< 0.001). Logistic regression analysis did not show a statistically significant association between CSCR and ischemic heart disease ( P =0.550), hypertension ( P =0.750), and hyperopia ( P =0.467). No pregnant female was present in our study sample, limiting our ability to assess its possible association with CSCR.

**Table 1:** Frequency of the investigated variables among cases and controls and odds ratio of central serous chorioretinopathy.


	orange**Case group, n (%)**	orange**Control group, n (%)**	orange**OR (95% Cl)**	orange**P-value**
Gender	Male	14 (35.9%)	44 (55.0%)	2.18 (0.99-4.80)	0.052
	Female	25 (64.1%)	36 (45.0%)	
Age		40.69 ± 7.71	40.58 ± 9.93	1.0 (0.961-1.04)	0.944
Alcohol	No	28 (71.8%)	80 (100.0%)	62.0	< 0.001
	Yes	11 (28.2%)	0 (0.0%)	
Smoking	No	27 (69.2%)	72 (90.0%)	4.0 (1.47-10.85)	0.006
	Yes	12 (30.8%)	8 (10.0%)	
Corticosteroid use	No	22 (56.4%)	72 (90.0%)	6.95 (2.64-18.28)	< 0.001
	Yes	17 (43.6%)	8 (10.0%)	
Coronary heart disease	No	39 (100.0%)	77 (96.2%)	- 0.550
	Yes	0 (0.0%)	3 (3.8%)	
Hypertension	No	34 (87.2%)	68 (85.0%)	0.83 (0.27-2.55)	0.750
	Yes	5 (12.8%)	12 (15.0%)	
Hyperopia	No	30 (76.9%)	68 (85.0%)	1.70 (0.40-7.09)	0.467
	Yes	9 (23.1%)	12 (15.0%)	
Pregnancy	No	39 (100.0%)	80 (100.0%)	- -
	Yes	0 (0.0%)	0 (0.0%)	
Psychological stress	No	5 (12.8%)	53 (66.3%)	13.34 (4.68-38.02)	< 0.001
	Yes	34 (87.2%)	27 (33.7%)	
	
	
white<bcol>6</ecol>OR, odds ratio; CI, confidence interval

##  Discussion

This case-control study aimed to investigate the possible association of CSCR with major social factors (smoking, alcohol consumption), comorbidities (hyperopia, ischemic heart disease, systemic hypertension), medications (corticosteroids), and psychological factors (stress) in an Iranian population. The results of the multivariate analysis indicated that smoking, alcohol consumption, corticosteroid use, and psychological stress were significantly more frequent in CSCR patients than in controls. However, no significant difference was detected regarding age, gender, hyperopia, ischemic heart disease, and systemic hypertension.

In the present study, the mean age at diagnosis was 40 ± 7.71. The mean age of CSCR patients in our study is consistent with, but slightly lower than, most of the previous studies, while the predominant gender in our study, unlike nearly all previous reports, is female. In line with our results, Kitzmann et al. reported a mean age of 41 among 74 CSCR patients. However, most studies have reported the mean age of patients to range from 41 to 45^[14]^. A recent large-scale study on 811 CSCR cases found a mean age of 46.8 ± 9.7, slightly higher than previous reports^[11]^.

The female-to-male ratio among our cases was 1.79:1 showing a female preponderance, unlike most studies in the literature. The reported male-to-female ratios vary across different populations, ranging from 2.7:1 to 7:1^[8]^. We believe that due to the consecutive, single-center method of patient recruitment and our study's relatively small sample size ( n =39), the female-to-male ratio might have accidentally deviated a bit from the previously reported numbers.

Smoking and tobacco smoke exposure have links to numerous ocular disorders, such as cataracts, dry eye, age-related macular degeneration, and Graves' ophthalmopathy^[15]^. Our results may add weight to the argument that smoking may be associated with CSCR risk (OR: 4.0, 95 % CI: 1.47-10.85, P =0.006). It was first suggested as a possible risk factor for CSCR by Spaide et al.^[16]^. As a preventable risk factor, understanding the extent of its possible association with CSCR is important. However, data remains inconsistent; some studies, like ours, confirm such an association^[1,5,11]^, and some do not^[2,12,17]^. The primary suggested pathogenesis of smoking in CSCR involves nicotine effects on choroidal blood flow autoregulation. Nicotine exposure can impair nitric oxide-induced vascular dilation, which, along with its facilitation of norepinephrine-induced vasoconstriction, could disturb the choroidal blood flow and give rise to CSCR^[18]^. Nicotine administration in mouse models increased the size and vascularity of choroidal neovascularization, and when combined with platelet-derived growth factor (PDGF), it resulted in hypertrophy of choroidal vascular smooth muscle^[19,20]^.

There is still uncertainty about whether alcohol consumption could play a role in the pathogenesis of CSCR^[2,4]^. Haimovici et al. found a significant association between alcohol consumption and the occurrence of CSCR^[18]^. A systematic review and meta-analysis of 17 studies confirmed alcohol consumption as a significant risk factor^[4]^. Ethanol is converted to acetaldehyde in hepatocytes shortly after ingestion, which may exert vasodilatory effects and elevate choroidal blood flow, resulting in transient thickening; the subfoveal choroidal thickness after oral ethanol intake may be greater than that following water consumption^[21]^. Like nicotine, alcohol, at higher doses, may impair nitric oxide-induced vasodilation, enhance norepinephrine-induced vasoconstriction, and increase arteriolar hydrostatic pressure^[18]^. Although some studies have not observed a significant association between alcohol consumption and CSCR^[5,12]^, our results are indicative of such an association ( P< 0.001). This heterogeneity may be related to differences in response to alcohol consumption and detoxification in each patient. In addition, accurate data on the amount and the period of alcohol consumption in both cases and controls are lacking. Further detailed investigations on this potential association are warranted in laboratory and clinical settings.

Several studies have investigated the association between CHD and CSCR; the results are still open to discussion. Some studies reported a positive association^[22]^, while others did not^[5,11]^. Hemostasis imbalance in patients with CHD may be the primary pathomechanism, along with abnormal fibrin deposition and increased serum PAI-1 levels^[5]^. Furthermore, some have found a reverse association; for example, a population-based study found that male patients with CSCR had a significantly higher CHD rate than those without CSCR, suggesting that CSCR may be a potential risk factor for the development of CHD in men^[23]^. We found no significant association between CHD and CSCR, which may suggest that, if present, such an association may have been too weak to be detected in our sample.

Although systemic hypertension can theoretically contribute to CSCR by inducing vasoconstriction in choriocapillaris and consequent compensatory outer choroidal hyperpermeability, its association with CSCR is still debated. A meta-analysis found hypertension to be a significant risk factor for CSCR (OR=1.7, P =0.0002)^[4]^. On the other hand, some recent studies found no significant association^[2][5,11]^. In the present study, the distribution of hypertensive patients was nearly equal in both groups. There was no significant association between hypertension and CSCR ( P =0.750). In this context, a limitation of our study was allocating patients to the “hypertensive” subgroup by the presence of antihypertensive medications in their drug history. It had two drawbacks: i) patients' recall bias when asked about their medications and ii) possible undiagnosed/uncontrolled hypertension. Further prospective studies with accurate blood pressure measurement and classification are warranted to avoid patients' recall bias and achieve more valid, consistent results.

Several recent studies have pointed out hyperopia as a risk factor for CSCR^[1,5,11][24]^. Hyperopia accompanied by increased choroidal thickening and reduced axial length may contribute to CSCR development^[1]^. A case-control study revealed that CSCR would more frequently involve hyperopic/emmetropic eyes with short axial lengths than myopic eyes with long axial lengths, highlighting the importance of anatomical parameters in CSCR pathogenesis^[24]^. Ersoz et al. found that each diopter increase in hyperopic change in the spherical equivalent refractive error was associated with a 24.6% increased risk of developing CSCR^[11]^. However, we failed to find any significant association between hyperopia and CSCR.

Many previous studies have confirmed corticosteroid use as a significant risk factor for CSCR^[1,4,25,26,27]^, but the underlying mechanism is still debated. Long-term steroid use can affect choroidal vasculature, inducing hyperpermeability and choroidal circulation decompensation^[5]^; other proposed mechanisms include steroid-induced platelet aggregation, microthrombus formation, and increased blood viscosity and vasoconstriction^[26]^. A meta-analysis confirmed this association; their subgroup analysis with respect to participants' ethnicity did not support this correlation in Asian people but confirmed it in the white population^[4]^. A population-based study in Taiwan also confirmed this association with oral corticosteroids, but not in a dose-dependent fashion^[27]^. Our study also found a significant relationship between corticosteroid use and CSCR in the Iranian population. This study was underpowered to perform subgroup analysis regarding the associated risk of each administration route.

Psychological factors have long been associated with CSCR; in 1983, Yannuzzi was the first to ascribe a portion of CSCR pathogenesis to type A personality through a presumed “sympathetic discharge”^[28]^. Recent evidence suggests that personality “traits” – rather than types – may be better at characterizing at-risk individuals, e.g., aggressiveness and anxiety traits^[29]^. While negatively affecting one's relationship with others, such maladaptive personality traits can lead to higher levels of perceived stress, which, in turn, may exacerbate the mentioned negative traits – forming a vicious cycle^[29]^. At this point, sleep disorders and insomnia are also closely associated with stress level rises and can further intensify the neuroendocrinological responses to high-stress conditions^[29]^. Psychological stimuli may be involved in CSCR pathogenesis through alterations in the hypothalamic-pituitary-adrenal axis and circulating cortisol levels^[18,30,31]^. In the present study, the risk of developing CSCR in patients with high psychological stress was 13.34 times higher than in others ( P< 0.01).

The literature strongly supports an association between pregnancy and CSCR. Chatziralli et al. found a 4.8-fold increased risk of developing CSCR in pregnant patients than in controls^[5]^. Another case-control study reported this risk even higher (OR=8.47)^[11]^. Yu et al. reported that the mean gestational age at CSCR onset was 27.11 ± 2.09 weeks. It was more likely to develop in the third trimester and usually resolved within 1-2 months postpartum^[31,32]^. We could not include pregnant patients in the present study to explore this association. Increased endogenous cortisol levels during pregnancy could partially explain this elevated risk^[32]^. Moreover, vasomotor stress in pregnancy can potentially lead to choroidal vasculature dysfunction, acting as another mechanism for increasing the risk of CSCR^[33]^.

Some limitations apply to the present study. It was a consecutive recruiting, single-center study in a referral eye care center, so potential selection bias could not be overcome. Also, the sample size was relatively small, precluding some subgroup analyses. A fraction of the data was collected retrospectively by reviewing patients' medical records, which may be subject to potential recall or reporting bias. In addition, our study lacked a reliable evaluation of participants for maladaptive personality traits and sleep disorders, limiting its ability to characterize the psychological risk factors of CSCR in more depth. However, the main strengths of this study were assembling an age-matched control group and the multivariate analytic approach. Notably, although our cohort had a higher female-to-male ratio than previous studies, it still demonstrated some previously reported risk factors. Further prospective investigations on the extent of the associations, especially for preventable risk factors, addressing the abovementioned gaps and studies exploring the pathogenesis of CSCR are recommended.

To summarize, our study showed that smoking, alcohol consumption, corticosteroid use, and psychological stress were independent risk factors for CSCR. Identifying the associated risk factors can help us better understand the underlying mechanism of CSCR to prevent and treat the disease more effectively. Further detailed investigations on the extent of these associations are warranted.

##  Financial Support and Sponsorship

None.

##  Conflicts of Interest

None.
